# Vitamin D Status in the Adult Population of Romania—Results of the European Health Examination Survey

**DOI:** 10.3390/nu16060867

**Published:** 2024-03-16

**Authors:** Lăcrămioara Aurelia Brîndușe, Irina Eclemea, Andrea Elena Neculau, Maria Alexandra Cucu

**Affiliations:** 1 Department of Public Health and Management, University of Medicine and Pharmacy Bucharest, 020021 Bucharest, Romania; lacramioara.brinduse@umfcd.ro; 2 Quality Management Department, Emergency University Hospital Elias, 011461 Bucharest, Romania; irina.eclemea@spitalul-elias.ro; 3 Department of Fundamental, Clinical and Prophylactic Sciences, Transylvania University of Brasov, 56 Nicolae Bălcescu Street, 500019 Brasov, Romania; 4 Department of Social Medicine, University of Medicine and Pharmacy Bucharest, 020021 Bucharest, Romania; alexandra.cucu@umfcd.ro

**Keywords:** vitamin D, deficiency, health survey, adult, Romania

## Abstract

Background: Vitamin D deficiency is recognized as a general health condition globally and is acknowledged as a public health concern in Europe. In Romania, a national program of examination of the status of vitamin D for high-risk groups has demonstrated a vitamin D deficiency prevalence of 39.83%. No national data on the status of vitamin D in the general adult population are available to date. Methods: We used the framework of the European Health Examination Survey to analyze vitamin D levels in a sample population of adults aged 25–64 years, from 120 family doctors’ patients lists, by using a sequential sampling method. Data were weighted to the Romanian population. Vitamin D deficiency was defined as 25(OH)D < 20 ng/mL. Results: In total, 5380 adults aged 25–64 years were included in this study. The overall prevalence of vitamin D deficiency is 24.8%. Predictors of vitamin D deficiency were found to be obesity, female sex, living in rural areas, lower education level, and lower socioeconomic status. Conclusions: Specific recommendations for vitamin D screening and supplementation should be issued for women by specialist boards. Further studies are needed to identify seasonal variation and to establish a correlation with nutritional surveys.

## 1. Introduction

Vitamin D deficiency is recognized as a general health condition globally [1,2,3] and is acknowledged as a public health concern at a European level [4,5], even in areas with high sun exposure such as the Mediterranean [6]. 

The authors of a systematic review and meta-analysis published in 2023 comprising a 20-year period and data from 7,947,359 participants from 81 countries concluded that, globally, vitamin D deficiency has a high prevalence [7]. 

Vitamin D is recognized for its roles in bone mineralization, calcium, and phosphorus intestinal absorption but also for its extra-skeletal implications in the immune system, cell growth, and metabolic system [8].

Additional effects of vitamin D deficiency have been observed in clinical trials, for example, the excess mortality rate; increased risk of infections, including severe COVID-19; and many other conditions and diseases such as muscle weakness, multiple sclerosis, diabetes, hypertension, metabolic syndrome, cancer, autoimmune diseases, and hip and vertebral fractures [2,7,9,10,11,12,13,14,15,16]. The role of vitamin D in cancer outcomes (relapse and fatal outcome) is promising [17,18,19,20].

Special attention has been paid to vitamin D deficiency in research regarding elderly populations. Experts conclude that supplementation of vitamin D, especially for those who are truly deficient, can offer significant benefits for bone health and extra-skeletal conditions, such as reduced risk of falling [21,22,23].

The COVID-19 pandemic has raised awareness of the importance of vitamin D status. An observational study on 191,190 patients showed that the positivity rate of SARS-CoV-2 was higher in patients with a deficiency of vitamin D [24]; however, a recently published review found no evidence of an association between historical vitamin D status and hospitalization or mortality due to COVID-19 and inconsistent results for any association between vitamin D and diagnosis of COVID-19 [25].

Vitamin D is scarce in foods. It is present in nature in small quantities, in plants such as fungi that contain ergosterol, the precursor of vitamin D2, and in animal foods such as fish liver oils and egg yolk; therefore, the daily allowance is difficult to ensure through diet alone [8]. Vitamin D is also produced in the skin through the effect of ultraviolet light (UV) of 7-dehydrocholesterol, which is isomerized into vitamin D3. Vitamin D3 is then converted in the kidneys into its bioactive form, 1α,25-dihydroxyvitamin D3, known as a prohormone, with levels regulated by the parathyroid hormone (PTH) and fibroblast growth factor 23 (FGF23) [26].

The recommended daily dose for vitamin D can be obtained through sunlight exposure, a supplemented diet (such as dairy products and cereals), or the administration of vitamin D [27]. 

One of the problems still being debated is the recommended daily dose of vitamin D intake. A review from 2020 summarizes the differences between recommendations from different organizations, underpinning the importance of administering higher doses for those at increased risk [28]. 

Risk factors associated with low vitamin D are less sun exposure, increased use of sunscreen, dark skin, obesity, gender, age, reduced physical activity, institutionalization, and poor socioeconomic status [1,3,29,30,31,32,33]. Certain medical conditions and medications can be associated with a higher risk of vitamin D deficiency [9]. 

It is generally agreed that high-risk populations can benefit from vitamin D correction but it is still a matter of debate among researchers whether offering supplements to the general population without a certified deficiency level can provide health benefits [12,13,21,27].

The adequate levels of 25(OH)D are also subject to debate. The minimum serum level to provide vitamin D sufficiency is considered by the National Academy of Medicine at ≥20 ng/mL and by the Endocrine Society at ≥30ng/mL [34]. The dosing of vitamin D should use a standardized method to allow comparability of the data [4,35].

Despite the escalation of the cut-offs for adequate levels of 25(OH)D, used especially for the purpose of demonstrating the extra-skeletal benefits of vitamin D (for cancer, immune disorders, and cardiovascular diseases), the recommended threshold for the serum level of 25(OH)D for the general population, adopted by most countries, is 20 ng/mL [21].

Most countries have national screening programs for measuring vitamin D levels in the general population or selected risk groups [1,36,37,38,39,40,41,42,43,44,45,46,47,48,49,50,51].

Monitoring the trends in vitamin D levels in the general population can provide an image of the health status and effects of measures such as vitamin D supplementation programs if in place, either through food fortification or through the administration of supplements [29].

Romania, a developed central eastern European country with a low per capita health expenditure, less than half of the EU average, is facing difficulties in implementing sustainable preventative programs and, to date, it has no data on the vitamin D status of the general population [52]. Since 2019, the Ministry of Health has established a National Program for Evaluation of the status of vitamin D through the determination of 25OHD for high-risk groups. According to the recommendations of the national guidelines endorsed by the Ministry of Health, risk groups considered for the program are adults presenting at the hospital with health conditions, pregnant and breastfeeding women, newborns, and children [53]. The results from the 2022 database of tests of the health program show a proportion of 39.83% of adults with a level of vitamin D deficiency under 20 ng/mL, of whom 13.4% had a vitamin D supplementation when tested [54]. This screening was an opportunistic one, with the population being selected from 16 hospitals spread all over the country; therefore, the data cannot be generalized to the population of Romania.

The European Health Examination Survey (EHES Romania) offered Romania the opportunity to determine the prevalence of major risk factors for non-communicable diseases. One of the main objectives of the survey was to document health policies in terms of health promotion, prevention, and monitoring of chronic diseases. 

Among the tested risk factors are weight, height, abdominal circumference, hip circumference, history of chronic diseases and chronic medication, smoking, alcohol, total lipid profile, glycemic profile, creatinine, hemoglobin, and vitamin D. 

Considering the importance of knowing the vitamin D status in the general adult population in Romania, the objective of this analysis is to evaluate the prevalence of vitamin D deficiency/insufficiency in a large population sample from the EHES Romania study 2023 and examine the association with selected demographic data and health risk factors. 

## 2. Methodology

### Study Design and Setting

Our analysis was performed under the methodological framework of the European Health Examination Survey (EHES), a European study that provides information about health and risk factors of the population to generate comparable data all over Europe but also for each country [55]. The study was undertaken in Romania between 5 August and 1 November 2022 with a target population of 9600 people and an estimated response rate of 50%.

The EHES is a complex sociomedical survey involving questionnaire data collection, blood sampling, and other anthropometric measurements. It follows the general methodology of EHES studies in Europe [49] but also has specific national parameters and data collection methodology [50]. It was coordinated by the National Institute of Public Health under Project PDP1/NT2311/13.05.2020, funded by the EEA Financial Mechanism 2014–2021, and implemented with the title “Strengthening the national network of primary health care providers to improve the health status of the population, children and adults (including vulnerable population)”.

The main objectives of EHES Romania are the evaluation of risk factors and associated chronic diseases, assessing health needs, and providing evidence for public health.

## 3. Study Population

The target population included female and male persons from all geographical regions of Romania aged between 25 and 64 years, who live in Romania. People in the target group are registered on the capitation lists of family doctors and can be either insured or uninsured in the healthcare system.

The following eligibility criteria were met by each sampled person: civilian status, non-institutionalized, male or female aged 25 years or older and not older than 64 years (with four age groups: 25–34, 35–44, 45–54, and 55–64), lives in Romania and meets the residency requirements at the country level, and are registered on the capitation lists of a family doctor as being insured or uninsured.

Persons who were excluded from the EHES are those who, at the time of implementation of the investigation, fulfilled one of the following conditions: citizens visiting the country for less than half of the last 12 months (tourists, in the country to see friends/relatives, etc.); citizens from certain professional categories (military, officers of public order and national security, etc.) who are registered on the lists of the health insurance fund Ministry of Defense Insurance House; and citizens who are institutionalized—including people living in hospitals, prisons, retirement homes, and other such institutions, which were not sampled in the EHES.

The parameters included in this study are as follows: age, sex, place of residence, level of education, income, lifestyle such as smoking, alcohol consumption, level of physical activity, anthropometric measurements (weight, height, waist circumference, and hip circumference), blood pressure (BP), timed stand chair test, blood tests, total cholesterol (total Chol), HDL cholesterol (HDL-Chol), LDL cholesterol (LDL-Chol), glycemia, full blood count (FBC), total calcium, gamma-glutamyl transferase (GGT), glycated hemoglobin (HbA1c), sodium, potassium, alanine transaminase (ALAT), triglycerides (TG), vitamin D levels, blood urea, T4, and TSH. The standard for anthropometric measurements is in accordance with the EHES study methodology [56]. The laboratory method used to determine 25(OH)D serum levels was chemiluminescence with immunoassay of small particles. Reference values for all blood parameters tested are summarized in the Supplementary Materials (Table S1).

The level of income was quantified according to the basic living expenses per family with two adults and children calculated for 2023 as per the Friedrich Ebert Stiftung and Syndex methodology [57]. The questionnaire was delivered face to face. 

To ensure the quality of data collection, all personnel selected were trained according to the standards of the EHES Romania protocol [58]. A pilot study of 200 respondents was used to validate the procedures.

**Figure 1:**
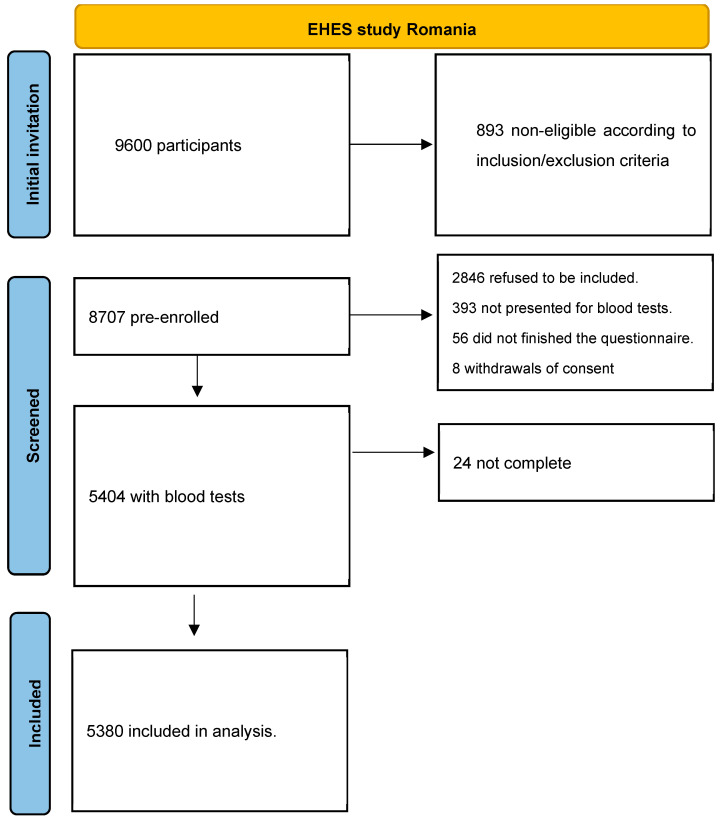
Selection of study population of EHES Romania.

The selection process is summarized in the following figure (Figure 1).

### 3.1. Selection of Participants

EHES Romania refers to a sampling based on capitation lists of family doctors. The sampling of potential participants in the EHES took place in two stages. In the first stage, data on 120 family doctors were randomly extracted by the method of probabilistic selection, proportional to the size of the unit, from the list of family doctors (who were listed as primary sampling units) from the NIH. Subsequently, in the second step, potential eligible participants in the EHES were selected from each family doctor’s capitation list (which functioned as secondary sampling units). The stratification of the sample of participants was performed based on two dimensions: the development region (there are eight such regions in Romania) and the urbanization level (rural/urban). In our study, we employed a design-based inference, with data from the sample being weighted to represent the structure of the Romanian population.

### 3.2. Data Collection Method

Interview operators (nurses) and family doctors ensured data collection. Both categories were instructed by the national coordination team on methodological procedures. The EHES questionnaire (in physical format, on paper) was applied by data collection operators (nurses) and the clinical evaluation was performed by family doctors.

The results of the questionnaire-based survey as well as of the other medical examinations were added to an integrated database. The database included individual-level measurement data from the EHES (including a questionnaire-based survey), data quality information, and personal (anonymized) information of each survey respondent. 

Each respondent was given a unique ID through which the data collected through the questionnaire could be united with the results of anthropometric measurements and other clinical examinations and laboratory tests.

A single consent form was used for all stages of participation, namely, questionnaire application, blood pressure measurement, anthropometric indicators, chair stand test, and blood sampling and the participant ticked only those stages for which he or she expressed his or her agreement to participate in the process.

### 3.3. Data Analysis

Data analysis was performed with SPSS 29.0 software (Statistical Package for Social Sciences; IBM Corp. Released 2022. IBM SPSS Statistics for Windows, Version 29.0. Armonk, NY, USA: IBM Corp). To adjust the sample for different sampling probabilities, a weighting factor was used to correct deviations in the sample from the Romanian population structure (according to the Romanian structure population from mid-July 2022). The weighting factor considered the sex, age group, residence area, and region. Moreover, the weighting factor included the selection probability by using the capitation list of family doctors. 

The distribution of continuous variables was analyzed, and the means and standard deviations were presented. The *t*-test was used to compare the means of continuous variables by sex. The qualitative data were presented as percentages (all results were weighted) and the chi-square was used to analyze the differences by sex. 

We established the serum 25(OH)D cut-offs according to laboratory references (<20 ng/mL = vitamin D deficiency; 20–30 ng/mL = vitamin D insufficiency; and >30 ng/mL = vitamin D sufficiency). We calculated the prevalence of vitamin D deficiency, insufficiency, and sufficiency by using the participants’ characteristics (age group, residence area, income, education level, behaviors such as alcohol consumption and smoking, ponderal status, associated diseases, and month of vitamin D assessment). The characteristics of patients were further analyzed in terms of their association with vitamin D deficiency and vitamin D insufficiency by using logistic regression models and the odds ratio (OR) and 95% CI were presented. 

We assessed the vitamin D predictors by using a sex-specific multiple linear regression. Firstly, the known or suspected determinants (age group, residence area, income, education level, behaviors such as alcohol consumption and smoking, ponderal status, associated diseases, and month of vitamin D assessment) of 25(OH)D concentrations were included in a linear model. Backward selection was performed to identify predictors of 25(OH)D concentration. If a category was significantly (*p* < 0.05) associated with vitamin D concentration, the variable was included in the final model (a multiple linear regression model). For the sex-specific final model, the age group, residence area, alcohol consumption, ponderal status, and month of vitamin D assessment were identified as predictors of vitamin D concentration in the male population and smoking, alcohol consumption, education, income, ponderal status, and month of vitamin D in the female population.

A *p*-value < 0.05 based on two-sided tests was considered statistically significant. 

### 3.4. Ethical Approval

This study has the approval of the institutional review board of the National Institute of Public Health nr 2857 from 17 February 2022.

All participants in the EHES received information on the aims and objectives of the survey, the medical examinations conducted, and data protection both in the forwarding letter and orally from their GPs or nurses. All participants signed the informed consent form (mandatory) before participating in the data collection process. 

The data collection teams explained the legal aspects of informed consent in simple language to participants, asked each participant to read the form, and made sure that they understood the key content. The participants had the opportunity to ask questions at any time during the visit to the medical offices/offices where the medical examinations were performed. Participants were also provided with contact numbers for further questions, even after the examination center visit.

## 4. Results

The descriptive characteristics of the study population, by sex, are presented in Table 1. There were no differences in average age and gender distribution. There is a significantly higher proportion of male participants in rural areas (*p* = 0.001), with a low level of education (*p* < 0.001) and higher income (*p* < 0.001) compared to women. The average body mass index is significantly higher in men (*p* < 0.001), with a statistically significantly higher prevalence (*p* < 0.001) of being overweight (40.3% vs. 31.8%) and obese (34.6% vs. 30.6%) among men compared to women.

The prevalence of smoking among men (29.5%) is significantly higher than among women (13.9%). Moreover, the prevalence of alcohol consumption differed according to the gender of study participants (*p* < 0.001), with a higher frequency of alcohol consumption and a significantly higher prevalence of binge drinking (*p* < 0.001) among men (51%) compared to women (13%). The prevalence of diabetes differs significantly depending on gender (*p* < 0.001), being 7.1% in men and 4.2% in women.

Mean values of 25(OH)D are significantly higher (*p* < 0.001) in men (28.0 ± 9.8) than in women (25.2 ± 8.9).

The characteristics of patients according to vitamin D status are shown in Table 2. The prevalence of vitamin D deficiency and insufficiency was higher in women (29.4% vs. 20.2% and 46.1% vs. 42.5%, respectively). The higher prevalence of vitamin D deficiency is found in urban areas (26.5% vs. 22.7% in rural areas) and in people with low education levels (25.9% vs. 23.2% in those with higher education levels). The prevalence of vitamin D deficiency and insufficiency is elevated among people with obesity (29.5% and 44.9%, respectively). Among participants who drank alcohol, the prevalence of vitamin D deficiency and insufficiency was lower than among those who never drank alcohol (20.2% vs. 30.0% and 41.2% vs. 45.4%, respectively).

The prevalence of vitamin D deficiency and insufficiency in women with hypertension, diabetes mellitus, and hypercholesterolemia was higher than in men.

The relationship between factors associated with vitamin D status was assessed based on OR, with OR levels of vitamin D deficiency (25(OH)D < 20 ng/mL) or insufficiency (25(OH)D 20–30 ng/mL) being presented in Table 3. In the entire study population, rural residence, education, income, smoking, and alcohol consumption had lower odds of vitamin D deficiency and alcohol consumption, smoking, hypercholesterolemia, and binge drinking had lower odds of vitamin D insufficiency. Among women, there were lower rates of vitamin D deficiency and insufficiency for education, income, and alcohol consumption and hypercholesterolemia had lower odds of vitamin D insufficiency. Obesity had greater odds of vitamin D deficiency and insufficiency for the entire sample included in the study. In men, obesity had higher odds of vitamin D insufficiency. In women, rural residence and obesity had higher odds for vitamin D insufficiency and obesity for vitamin D deficiency. Underweight was also associated with higher odds of either vitamin D deficiency or insufficiency.

Compared to August, samples collected in September and October had higher odds of vitamin D deficiency and insufficiency.

In men, the multiple stepwise logistic regression showed that rural residence and frequent alcohol consumption were significantly associated with higher 25(OH)D concentration, whereas being obese and an autumn month of vitamin D assessment were associated with lower 25(OH)D concentration (Table 4).

In women, occasional alcohol consumption and high education level were significantly associated with higher 25(OH)D concentration. Obesity and an autumn month of vitamin D assessment were associated with lower 25(OH)D concentration (Table 4).

## 5. Discussion

Our analysis investigated the status of vitamin D in an adult population sample of 5380, randomly selected to represent the Romanian population aged from 25 to 64 years. It showed that vitamin D deficiency, defined as <20 ng/mL, has a prevalence of 24.8% and vitamin D insufficiency, defined as 20–30 ng/mL, has a prevalence of 44.3%. The prevalence is below the figures reported from studies in France (34.6%) [59], Germany (61.6%) [60], Greece (54.4%) [61], and Russia (34%) [48]. Furthermore, a UK study showed a prevalence of vitamin D under 20 ng/mL of 37.8%, which is higher than in Romania, but it defines this as insufficiency [45]. 

Comparable deficiency rates were observed in a Norwegian prevalence study (24.7%) [46]. It is worth noting that the Norwegian study had a similar collection period that was performed in counties from the northern part of the country, with little sun exposure, in an older population group (40–69). It is also important to note that in Nordic countries, there is a general recommendation for a daily intake of vitamin D of 400 UI for adults up to 75 years of age and 800 UI for those aged over 75. Food supplementation with vitamin D from dairy products is also in place in Norway [46].

The overall lower rates of vitamin D deficiency seen in our country can be linked to the fact that elderly people (over 65) were not included in this study. Another possible explanation for the lower rates is the period of data collection from the EHES study, between August and October, with no inclusion of the winter season. Moreover, since during the month of August, which was considered the reference month for the analysis, a smaller number of patients were enrolled, we obtained a reduced statistical power. According to the National Administration of Meteorology, the climate of Romania is temperate continental with four distinct seasons [62]. During summer, sun exposure is generally high in most of the regions [63]. During spring and autumn, there is moderate sun exposure, whereas in winter, sun exposure is reduced. In our study, the odds of vitamin D deficiency are five times higher in September and nearly eight times higher in October than in August.

The reduced exposure to ultraviolet radiation can also account for the higher prevalence of vitamin D deficiency in urban areas (26.5%) in comparison with rural ones (22.7%). Men living in rural areas have a 36% lower odds of vitamin D deficiency in comparison with those living in urban areas, probably due to a predominance of outdoor work, whereas women, by contrast, have higher odds in rural areas for vitamin D insufficiency.

Concerning the low prevalence of vitamin D deficiency observed in our study, it is also important to underline that the questionnaire did not evaluate the use of vitamin D supplements in the population tested. Nonetheless, a Romanian study from 2016, based on a nutritional questionnaire that estimated the dietary intake of vitamin D from foods, showed that vitamin D intake in the Romanian population is insufficient [64].

One of the significant results of our study is a higher prevalence of vitamin D deficiency in women (29.4%) in comparison with men (20.2%). Several studies also report higher prevalence in women [31,44,47], whereas some found dominance in men [39,45] and others found no sex-dependent differences [59]. A study in Germany found a higher prevalence of vitamin D deficiency in men during autumn and in women during winter, a result that is also supported by our study (12.53 times higher odds for vitamin D deficiency in men in October in comparison with August) [60]. 

In our study, educated women have 44% lower odds of deficiency and 28% lower odds of insufficiency in comparison with less-educated women. Socioeconomic status also has a notable impact on vitamin D levels. Women with a higher economic status have 48% lower odds of vitamin D deficiency and 26% lower odds of vitamin D deficiency.

Education and socioeconomic status of women as predictors of vitamin D status are important findings, useful for documenting the need for tailored educational campaigns for women regarding the importance of vitamin D. We consider this in light of the known impact of vitamin D deficiency in women of childbearing age on their pregnancies, with potential complications such as hypertension, gestational diabetes, negative perinatal outcomes, and first-trimester miscarriages [65,66,67,68]. This can also be reflected in child nutritional and orthopedic problems [66]. It also must be considered in post-menopausal women, who have an increased risk of fractures, with an almost 50% increase at concentrations of 25(OH)D < 15 ng/mL and an increase of 33% for each 10 ng/mL decrease in 25(OH)D [69].

Smoking status was found to be a negative predictor of vitamin D deficiency and insufficiency, especially in women. Contradictory results are reported in studies from Australia [39], the UK [45], and Norway [46]. The possible confounding effect in our study is due to the design of the questionnaire, which included occasional smokers under the same umbrella as daily smokers, most of the occasional smokers being women.

Different population groups have been shown to have a higher prevalence of vitamin D deficiency in our study. The obese group (as per BMI) has a higher-than-average overall vitamin D deficiency (29.5%). Obese women have a 2.49 higher odds of vitamin D deficiency than non-obese ones and obese men have 34% higher odds of vitamin D insufficiency in relation to those with normal weight. 

In the literature, vitamin D status was found to have a strong inverse association with visceral adiposity and a potential inverse association with insulin resistance [15]. 

In our study, a self-reported history of HBP, diabetes, and hypercholesterolemia were not associated with vitamin D deficiency. Moreover, hypercholesterolemic women showed lower odds of vitamin D insufficiency compared with those with normal cholesterol levels. This is a paradoxical finding of our study, considering the results of studies that have analyzed the influences between bone, glucose, and lipid metabolism and found that hypovitaminosis D predisposes people toward worsening lipid profiles [70]. This is possibly because of the underdiagnosis of cardiovascular and metabolic diseases for the age groups included in this study.

Considering data from our study but also actual data from the literature, it is difficult to draw conclusions about whether a supplementation program of vitamin D could be beneficial in terms of health outcomes for people with cardiometabolic diseases [11,12,13]. 

## 6. Conclusions

Although our data show a lower prevalence of vitamin D deficiency, a solid conclusion could not be drawn without a wider interval of data collection throughout the year to include seasonal variability, a population sample of over 65, and data on the nutritional intake of vitamin D.

Our data revealed a possible health risk group in obese women with a lower socioeconomic status and lower educational level living in rural areas, who were more likely to have low serum vitamin D levels. This suggests a need for the professional board of the Ministry of Health to issue a general recommendation for vitamin D status evaluation and correction in this category of population. Further studies are needed to identify seasonal variation and to establish correlation with nutritional surveys but also to assess the efficacy of interventions. 

### Limitations

Our study sample did not include people aged over 65 years; therefore, we cannot draw important conclusions on this risk group. Considering the recommendations of the international consensus of experts of 2023 that concluded that vitamin D supplements to restore inadequate 25(OH)D levels are the most effective and practical intervention in the aging population, we believe that, currently, evaluations can be realized on an individual basis as per the recommendations of prevention guidelines in adults published in 2023 by the National Institute of Health.

This study was not projected to analyze vitamin D deficiency as the only outcome of research and the questionnaire did not evaluate dietary patterns or vitamin D supplementation used in the study population. Furthermore, the period of data collection did not comprise all seasons; so, seasonal variability could not be identified. 

Dedicated research in this area should be designed and developed at the national level.

## Figures and Tables

**Table 1:** Descriptive characteristics of the participants, by sex.

Variable	Overall (*n* = 5380)	Male (*n* = 2315)	Female (*n* = 3065)	*p*-Value
25(OH)D (ng/mL) (mean ± SD)	26.6 ± 9.5	28.0 ± 9.8	25.2 ± 8.9	<0.001
Age (mean ± SD)	45.1 ± 10.9	44.8 ± 10.8	45.4 ± 11.0	0.063
Age group (%)				0.221
25–34 years	1012 (21.2)	422 (21.6)	590 (20.8)	
35–44 years	1465 (26.3)	650 (26.9)	815 (25.7)	
45–54 years	1637 (30.4)	714 (30.6)	923 (30.2)	
55–64 years	1266 (22.2)	529 (20.9)	737 (23.4)	
Residence (%)				0.001
Urban	2927 (53.2)	1283 (50.8)	1644 (55.7)	
Rural	2453 (46.8)	1032 (49.2)	1421 (44.3)	
Education (%)				<0.001
≤12	3118 (58.6)	1382 (61.3)	1736 (55.8)	
>12	2262 (41.4)	933 (38.7)	1329 (44.2)	
Income (%)				0.001
Low	3462 (63.7)	1406 (61.1)	2056 (66.4)	
Over basic living expenses	796 (14.9)	391 (16.2)	405 (13.4)	
Do not declare	1122 (21.4)	518 (22.7)	604 (20.2)	
BMI (kg/m^2^) (mean ± SD)	28.1 ± 5.9	28.6 ± 5.5	27.6 ± 6.2	<0.001
Underweight N (%)	103 (1.7)	14 (0.6)	89 (2.9)	<0.001
Normal N (%)	1616 (29.6)	557 (24.5)	1059 (34.7)	
Overweight N (%)	1904 (36.1)	933 (40.3)	971 (31.8)	
Obese N (%)	1741 (32.6)	805 (34.6)	936 (30.6)	
Smoking status (%)				<0.001
Non-smoker	3305 (78.7)	1170 (70.5)	2135 (86.1)	
Smoker	831 (21.3)	492 (29.5)	339 (13.9)	
Alcohol consumption (%)				<0.001
Never drink	2102 (36.5)	444 (19.2)	1658 (54.1)	
Drink occasionally	1854 (34.9)	800 (34.8)	1054 (34.9)	
Drink frequently	1392 (28.6)	1058 (46.0)	334 (11.0)	
Binge drinking	1124 (37.3)	943 (51.0)	181 (13.0)	<0.001
Comorbidities (%)				
HBP *	1342 (24.2)	614 (25.0)	728 (23.3)	0.170
T2DM **	310 (5.6)	177 (7.1)	133 (4.2)	<0.001
H-Chol ***	1152 (21.0)	540 (22.2)	612 (19.8)	0.042
Month				0.270
August	50 (1.0)	16 (0.9)	34 (1.0)	
September	3563 (66.1)	1508 (65.2)	2055 (67.1)	
October	1767 (32.9)	791 (33.9)	976 (31.9)	

Results are weighted, except for the number of cases. * HBP = high blood pressure, ** T2DM = diabetes mellitus type 2, *** H-Chol = hypercholesterolemia, BMI = body mass index (kg/m^2^)—Underweight < 18.5; Normal weight 18.5–24.9; overweight 25–29.9; obese ≥ 30.

**Table 2:** Prevalence of 25(OH)D status, by sex.

Variables	Vitamin D Deficiency (<20 ng/mL)			Vitamin D Insufficiency (20–30 ng/mL)			Vitamin D Sufficiency (>30 ng/mL)		
	Overall	Male	Female	Overall	Male	Female	Overall	Male	Female
Overall	24.8	20.2	29.4	44.3	42.5	46.1	30.9	37.3	24.5
Age group									
25–34 years	25.5	22.5	28.6	44.8	43.6	46.2	29.7	34.0	25.2
35–44 years	23.7	19.7	27.9	45.6	43.8	47.5	30.7	36.5	24.6
45–54 years	24.7	19.3	30.3	43.8	41.4	46.3	31.5	39.3	23.4
55–64 years	25.4	19.8	30.3	42.9	41.3	44.3	31.8	38.9	25.3
Residence									
Urban	26.5	23.0	29.8	43.5	42.7	44.3	30.0	34.3	25.9
Rural	22.7	17.3	28.8	45.2	42.4	48.4	32.1	40.3	22.8
Education									
≤12	25.9	19.8	32.6	44.1	42.2	46.4	30.0	38.0	21.0
>12	23.2	20.9	25.3	44.5	43.1	45.8	32.2	36.0	28.9
Income									
Low	25.7	19.5	31.5	45.1	43.8	46.4	29.1	36.7	22.0
Over basic living expenses	21.2	19.9	22.7	44.9	43.1	47.0	33.9	37.0	30.2
Do not declare	24.4	22.3	26.8	41.4	38.7	44.5	34.2	39.1	28.7
BMI *									
Underweight	29.3	26.7	29.9	42.7	26.7	46.3	28.0	46.7	23.9
Normal	23.7	20.5	25.9	42.9	39.3	45.5	33.4	40.2	28.6
Overweight	21.0	17.8	25.3	45.1	43.4	47.2	33.9	38.8	27.5
Obese	29.5	22.5	37.4	44.9	43.9	45.9	25.7	33.6	16.6
Smoking status									
Never smoker	25.1	19.1	29.6	45.3	43.1	46.9	29.6	37.8	23.5
Smoker > 1 year	23.7	23.0	25.2	41.3	39.3	45.1	35.0	37.7	29.7
Alcohol consumption									
Never	30.0	24.1	32.1	45.4	41.9	46.7	24.6	34.1	21.2
Occasionally	23.0	19.6	26.3	45.7	46.0	45.4	31.3	34.4	28.3
Frequently	20.2	18.9	25.7	41.2	40.1	45.6	38.6	40.9	28.7
Binge drinking	21.2	20.0	29.9	40.4	39.9	43.8	32.3	35.9	28.7
Comorbidities									
HBP **	25.3	21.0	30.0	43.0	40.8	45.3	31.7	38.2	24.6
T2DM ***	25.9	23.4	30.3	44.4	43.9	45.5	29.6	32.7	24.2
H-Chol ****	25.5	21.1	30.5	24.6	20.0	29.1	33.1	37.4	28.2
Month									
August	6.5	4.8	8.0	41.3	23.8	56.0	52.2	71.4	36.0
September	23.5	19.5	27.4	43.2	39.4	46.9	33.3	41.1	25.8
October	27.9	22.0	34.3	46.6	48.8	44.3	25.5	29.2	21.5

** HBP = high blood pressure, *** T2DM = diabetes mellitus type 2, **** H-Chol = hypercholesterolemia, BMI * = body mass index.

**Table 3:** Association between participants’ characteristics and low vitamin D status.

Variables	OR of Vitamin D Deficiency			OR of Vitamin D Insufficiency		
	Overall	Male	Female	Overall	Male	Female
Age group						
25–34 years	1	1	1	1	1	1
35–44 years	0.89 (0.72–1.12)	0.81 (0.59–1.11)	0.99 (0.72–1.38)	0.99 (0.81–1.20)	0.94 (0.72–1.21)	1.06 (0.79–1.42)
45–54 years	0.91 (0.74–1.13)	0.74 (0.54–1.01)	1.14 (0.83–1.56)	0.92 (0.76–1.11)	0.82 (0.63–1.05)	1.09 (0.82–1.45)
55–64 years	0.93 (0.74–1.17)	0.77 (0.55–1.08)	1.06 (0.76–1.47)	0.89 (0.73–1.10)	0.83 (0.63–1.09)	0.96 (0.71–1.30)
Residence						
Urban	1	1	1	1	1	1
Rural	0.80 (0.69–0.93)	0.64 (0.51–0.80)	1.10 (0.88–1.37)	0.97 (0.85–1.11)	0.85 (0.71–1.01)	1.24 (1.02–1.52)
Education						
≤12	1	1	1	1	1	1
>12	0.83 (0.71–0.97)	1.11 (0.88–1.39)	0.56 (0.45–0.71)	0.94 (0.82–1.07)	1.08 (0.89–1.30)	0.72 (0.59–0.88)
Income						
Low	1	1	1	1	1	1
Over basic living expenses	0.70 (0.56–0.88)	1.01 (0.74–1.37)	0.52 (3.37–0.73)	0.85 (0.71–1.03)	0.98 (0.76–1.25)	0.74 (0.56–0.98)
Do not declare	0.81 (0.67–0.97)	1.07 (0.82–1.39)	0.66 (0.50–0.86)	0.78 (0.66–0.92)	0.83 (0.67–1.04)	0.74 (0.57–0.95)
BMI (kg/m^2^) *						
Underweight	1.46 (0.81–2.64)	1.21 (0.34–4.28)	1.33 (0.67–2.62)	1.19 (0.69–2.05)	0.64 (0.18–2.23)	1.19 (0.64–2.21)
Normal	1	1	1	1	1	1
Overweight	0.88 (0.73–1.06)	0.89 (0.67–1.19)	1.02 (0.78–1.33)	0.88 (0.72–1.06)	1.15 (0.91–1.44)	1.08 (0.85–1.37)
Obese	1.63 (1.34–1.97)	1.32 (0.99–1.75)	2.49 (1.88–3.30)	1.37 (1.15–1.62)	1.34 (1.06–1.71)	1.74 (1.34–2.27)
Smoking status (ref. ** non-smoker)						
Smoker	0.80 (0.65–0.99)	1.21 (0.91–1.59)	0.67 (0.48–0.96)	0.77 (0.64–0.93)	0.92 (0.73–1.16)	0.75 (0.55–1.03)
Alcohol consumption						
Never drink	1	1	1	1	1	1
Drink occasionally	0.60 (0.50–0.72)	0.81 (0.59–1.09)	0.62 (0.49–0.78)	0.79 (0.67–0.93)	1.01 (0.84–1.41)	0.73 (0.59–0.91)
Drink frequently	0.43 (0.35–0.52)	0.65 (0.48–0.87)	0.59 (0.41–0.84)	0.58 (0.49–0.68)	0.79 (0.62–1.02)	0.72 (0.52–0.99)
Binge drinking	0.81 (0.66–0.98)	0.96 (0.75–1.23)	1.27 (0.79–2.03)	0.74 (0.63–0.88)	0.78 (0.64–0.95)	1.05 (0.68–1.61)
Comorbidities						
HBP ***	0.99 (0.83–1.19)	1.01 (0.79–1.31)	1.01 (0.79–1.32)	0.93 (0.80–1.09)	0.92 (0.75–1.13)	0.97 (0.77–1.23)
T2DM ****	1.10 (0.79–1.52)	1.32 (0.86–2.01)	1.07 (0.62–1.85)	1.05 (0.78–1.40)	1.18 (0.83–1.70)	0.99 (0.60–1.66)
H-Chol *****	0.95 (0.79–1.14)	1.05 (0.81–1.37)	0.87 (0.67–1.14)	0.84 (0.72–0.99)	0.97 (0.78–1.20)	0.73 (0.57–0.93)
Month						
August	1	1	1	1	1	1
September	5.10 (1.60–16.29)	7.93 (0.91–69.15)	4.05 (0.97–16.93)	1.60 (0.87–2.93)	2.61 (0.96–7.08)	1.20 (0.52–2.79)
October	7.91 (2.47–25.34)	12.53 (1.43–109.80)	6.05 (1.44–25.46)	2.26 (1.22–4.17)	4.55 (1.66–12.41)	1.36 (0.58–3.19)

** ref. = reference. *** HBP = high blood pressure, **** T2DM = diabetes mellitus type 2, ***** H-Chol = hypercholesterolemia, BMI * = body mass index (kg/m^2^). Results were obtained from a linear logistic model.

**Table 4:** Predictors of vitamin D concentration (ng/mL).

	Males			Females		
	B	SE	*p*-Value	B	SE	*p*-Value
Intercept	32.47	2.26	<0.001	29.54	1.88	<0.001
Age (* ref. 25–34 years)						
35–44 years	0.92	0.58	0.11	-	-	-
45–54 years	1.38	0.57	0.02	-	-	-
55–64 years	1.17	0.63	0.06	-	-	-
Residence (ref. urban)						
Rural	1.29	0.40	0.001	-	-	-
Smoking (ref. non-smokers)						
Smokers	-	-	-	1.02	0.58	0.08
Alcohol (ref. never)						
Occasionally	0.56	0.56	0.32	1.19	0.44	0.006
Frequently	1.32	0.54	0.01	0.75	0.69	0.28
Education (ref. ≤ 12 years)						
High (>12 years)	-	-	-	1.40	0.44	0.002
Income (ref. < low)						
High	-	-	-	0.27	0.62	0.67
NA	-	-	-	0.91	0.50	0.07
Ponderal status (ref. normal)						
Underweight	4.43	2.59	0.08	−1.72	1.32	0.19
Overweight	0.08	0.51	0.87	−0.58	0.49	0.24
Obese	−1.32	0.54	0.01	−2.62	0.50	<0.001
Month (ref. August)						
September	−5.83	2.16	0.007	−4.37	1.85	0.02
October	−7.54	2.17	<0.001	−5.67	1.87	0.003

* ref. = reference. Results were obtained from a sex-specific multiple regression model; B. = beta coefficient; SE. = standard error.

## Data Availability

The data presented in this study are available on request from the first author (lacramioara.brinduse@umfcd.ro).

## Supplementary Materials

The following supporting information can be downloaded at https://www.mdpi.com/article/10.3390/nu16060867/s1: Table S1: Normal ranges for laboratory values.

## References

[1] Holick M.F., Chen T.C. (2008). Vitamin D deficiency: A worldwide problem with health consequences. Am. J. Clin. Nutr..

[2] Amrein K., Scherkl M., Hoffmann M., Neuwersch-Sommeregger S., Köstenberger M., Tmava Berisha A., Martucci G., Pilz S., Malle O. (2020). Vitamin D deficiency 2.0: An update on the current status worldwide. Eur. J. Clin. Nutr..

[3] Hilger J., Friedel A., Herr R., Rausch T., Roos F., Wahl D.A., Pierroz D.D., Weber P., Hoffmann K. (2014). A systematic review of vitamin D status in populations worldwide. Br. J. Nutr..

[4] Cashman K.D., Dowling K.G., Škrabáková Z., Gonzalez-Gross M., Valtueña J., De Henauw S., Moreno L., Damsgaard C.T., Michaelsen K.F., Mølgaard C. (2016). Vitamin D deficiency in Europe: Pandemic?. Am. J. Clin. Nutr..

[5] Wahl D.A., Cooper C., Ebeling P.R., Eggersdorfer M., Hilger J., Hoffmann K., Josse R., Kanis J.A., Mithal A., Pierroz D.D. (2012). A global representation of vitamin D status in healthy populations. Arch. Osteoporos..

[6] Díaz-Rizzolo D.A., Kostov B., Gomis R., Sisó-Almirall A. (2022). Paradoxical suboptimal vitamin D levels in a Mediterranean area: A population-based study. Sci. Rep..

[7] Cui A., Zhang T., Xiao P., Fan Z., Wang H., Zhuang Y. (2023). Global and regional prevalence of vitamin D deficiency in population-based studies from 2000 to 2022: A pooled analysis of 7.9 million participants. Front. Nutr..

[8] DeLuca H.F. (2004). Overview of general physiologic features and functions of vitamin D. Am. J. Clin. Nutr..

[9] Edwards M.H., Cole Z.A., Harvey N.C., Cooper C. (2014). The global epidemiology of vitamin D status. J. Aging Res. Clin. Pract..

[10] Martineau A.R., Jolliffe D.A., Hooper R.L., Greenberg L., Aloia J.F., Bergman P., Dubnov-Raz G., Esposito S., Ganmaa D., Ginde A.A. (2017). Vitamin D supplementation to prevent acute respiratory tract infections: Systematic review and meta-analysis of individual participant data. BMJ.

[11] Barbarawi M., Kheiri B., Zayed Y., Barbarawi O., Dhillon H., Swaid B., Yelangi A., Sundus S., Bachuwa G., Alkotob M.L. (2019). Vitamin D Supplementation and Cardiovascular Disease Risks in More Than 83,000 Individuals in 21 Randomized Clinical Trials: A Meta-analysis. JAMA Cardiol..

[12] Pilz S., Trummer C., Theiler-Schwetz V., Grübler M.R., Verheyen N.D., Odler B., Karras S.N., Zittermann A., März W. (2022). Critical Appraisal of Large Vitamin D Randomized Controlled Trials. Nutrients.

[13] Hu C., Yang M. (2024). Trends of serum 25(OH) vitamin D and association with cardiovascular disease and all-cause mortality: From NHANES survey cycles 2001–2018. Front. Nutr..

[14] Dawson-Hughes B., Staten M.A., Knowler W.C., Nelson J., Vickery E.M., LeBlanc E.S., Neff L.M., Park J., Pittas A.G., D2d Research Group (2020). Intratrial Exposure to Vitamin D and New-Onset Diabetes among Adults with Prediabetes: A Secondary Analysis from the Vitamin D and Type 2 Diabetes (D2d) Study. Diabetes Care.

[15] Valer-Martinez A., Martinez J.A., Sayon-Orea C., Galvano F., Grosso G., Bes-Rastrollo M. (2019). Vitamin D and Cardio-Metabolic Risk Factors in Overweight Adults: An Overview of the Evidence. Curr. Pharm. Des..

[16] Win S.S., Camargo C.A., Khaw K.T., Lawes C.M.M., Sluyter J., Waayer D., Toop L., Scragg R. (2019). Cross-sectional associations of vitamin D status with asthma prevalence, exacerbations, and control in New Zealand adults. J. Steroid Biochem. Mol. Biol..

[17] Chandler P.D., Chen W.Y., Ajala O.N., Hazra A., Cook N., Bubes V., Lee I.-M., Giovannucci E.L., Willett W., Buring J.E. (2020). Effect of Vitamin D3 Supplements on Development of Advanced Cancer: A Secondary Analysis of the VITAL Randomized Clinical Trial. JAMA Netw. Open.

[18] Muñoz A., Grant W.B. (2022). Vitamin D and Cancer: An Historical Overview of the Epidemiology and Mechanisms. Nutrients.

[19] Holick M.F. (2023). The Death D-Fying Vitamin D_3_ for Digestive Tract Cancers—The p53 Antibody Connection. JAMA Netw. Open.

[20] Kanno K., Akutsu T., Ohdaira H., Suzuki Y., Urashima M. (2023). Effect of Vitamin D Supplements on Relapse or Death in a p53-Immunoreactive Subgroup with Digestive Tract Cancer: Post Hoc Analysis of the AMATERASU Randomized Clinical Trial. JAMA Netw. Open.

[21] Hu Y., Wang R., Mao D., Chen J., Li M., Li W., Yang X., Yang L. (2023). Vitamin D Status and Associated Factors of Older Adults in the Cross-Sectional 2015–2017 Survey. Nutrients.

[22] Giustina A., Bouillon R., Dawson-Hughes B., Ebeling P.R., Lazaretti-Castro M., Lips P., Marcocci C., Bilezikian J.P. (2023). Vitamin D in the older population: A consensus statement. Endocrine.

[23] Harse J.D., Zhu K., Bucks R.S., Hunter M., Lim E.M., Cooke B.R., Walsh J.P., Murray K. (2021). Investigating Potential Dose-Response Relationships between Vitamin D Status and Cognitive Performance: A Cross-Sectional Analysis in Middle- to Older-Aged Adults in the Busselton Healthy Ageing Study. Int. J. Environ. Res. Public Health.

[24] Kaufman H.W., Niles J.K., Kroll M.H., Bi C., Holick M.F. (2020). SARS-CoV-2 positivity rates associated with circulating 25-hydroxyvitamin D levels. PLoS ONE.

[25] Lin L.-Y., Mulick A., Mathur R., Smeeth L., Warren-Gash C., Langan S. (2022). The association between vitamin D status and COVID-19 in England: A cohort study using UK Biobank. PLoS ONE.

[26] Gallagher J.C., Rosen C.J. (2023). Vitamin D: 100 years of discoveries, yet controversy continues. Lancet Diabetes Endocrinol..

[27] Cashman K.D., Kiely M. (2011). Towards prevention of vitamin D deficiency and beyond: Knowledge gaps and research needs in vitamin D nutrition and public health. Br. J. Nutr..

[28] Kimball S.M., Holick M.F. (2020). Official recommendations for vitamin D through the life stages in developed countries. Eur. J. Clin. Nutr..

[29] Lips P., de Jongh R.T., van Schoor N.M. (2021). Trends in Vitamin D Status Around the World. JBMR Plus.

[30] Mendoza-Garcés L., Velázquez-Alva M.C., Cabrer-Rosales M.F., Arrieta-Cruz I., Gutiérrez-Juárez R., Irigoyen-Camacho M.E. (2021). Vitamin D Deficiency is Associated with Handgrip Strength, Nutritional Status and T2DM in Community-Dwelling Older Mexican Women: A Cross-Sectional Study. Nutrients.

[31] Yan X., Zhang N., Cheng S., Wang Z., Qin Y. (2019). Gender Differences in Vitamin D Status in China. Med. Sci. Monit..

[32] Bouillon R., Manousaki D., Rosen C., Trajanoska K., Rivadeneira F., Richards J.B. (2022). The health effects of vitamin D supplementation: Evidence from human studies. Nat. Rev. Endocrinol..

[33] Gallagher J.C., Sai A.J. (2010). Vitamin D insufficiency, deficiency, and bone health. J. Clin. Endocrinol. Metab..

[34] Holick M.F., Binkley N.C., Bischoff-Ferrari H.A., Gordon C.M., Hanley D.A., Heaney R.P., Murad M.H., Weaver C.M., Endocrine Society (2011). Evaluation, treatment, and prevention of vitamin D deficiency: An Endocrine Society clinical practice guideline. Med. J. Clin. Endocrinol. Metab..

[35] Sempos C.T., Vesper H.W., Phinney K.W., Thienpont L.M., Coates P.M. (2012). Vitamin D status as an international issue: National surveys and the problem of standardization. Scand. J. Clin. Lab. Investig. Suppl..

[36] Acherjya G.K., Ali M., Tarafder K., Akhter N., Chowdhury M.K., Islam D.U., Rahman M.H., Miah M.T. (2019). Study of Vitamin D Deficiency among the Apparently Healthy Population in Jashore, Bangladesh. Mymensingh Med. J..

[37] Akinlawon O.J., Lai C.Q., Noel S.E., Flanagan K.D., Tucker K.L. (2021). Risk Factors Associated with Vitamin D Status among Older Puerto Rican Adults. J. Nutr..

[38] Farhat K.H., Arafa M.A., Rabah D.M., Amin H.S., Ibrahim N.K. (2019). Vitamin D status and its correlates in Saudi male population. BMC Public Health.

[39] Gill T.K., Hill C.L., Shanahan E.M., Taylor A.W., Appleton S.L., Grant J.F., Shi Z., Dal Grande E., Price K., Adams R.J. (2014). Vitamin D levels in an Australian population. BMC Public Health.

[40] Hutchings N., Babalyan V., Heijboer A.C., Baghdasaryan S., Qefoyan M., Ivanyan A., Ackermans M.T., Formenti A.M., Lesnyak O., Giustina A. (2022). Vitamin D status in Armenian women: A stratified cross-sectional cluster analysis. Eur. J. Clin. Nutr..

[41] Jiang W., Wu D.B., Xiao G.B., Ding B., Chen E.Q. (2020). An epidemiology survey of vitamin D deficiency and its influencing factors. Med. Clin..

[42] Kamińska S., Pikala M., Dziankowska-Zaborszczyk E., Bielecki W., Rębowska E., Kozakiewicz K., Nadrowski P., Drygas W., Kwaśniewska M. (2020). Vitamin D—Dietary intake, supplementation and metabolic status of Polish adults. Int. J. Occup. Med. Environ. Health.

[43] Leão L., Rodrigues B.C., Dias P.T.P., Gehrke B., Souza T., Hirose C.K., Freire M.D.C. (2021). Vitamin D status and prevalence of hypovitaminosis D in different genders throughout life stages: A Brazilian cross-sectional study. Clinics.

[44] Lee M.J., Hsu H.J., Wu I.W., Sun C.Y., Ting M.K., Lee C.C. (2019). Vitamin D deficiency in northern Taiwan: A community-based cohort study. BMC Public Health.

[45] Lin L.Y., Smeeth L., Langan S., Warren-Gash C. (2021). Distribution of vitamin D status in the UK: A cross-sectional analysis of UK Biobank. BMJ Open.

[46] Petrenya N., Lamberg-Allardt C., Melhus M., Broderstad A.R., Brustad M. (2020). Vitamin D status in a multi-ethnic population of northern Norway: The SAMINOR 2 Clinical Survey. Public Health Nutr..

[47] Siddiqee M.H., Bhattacharjee B., Siddiqi U.R., MeshbahurRahman M. (2021). High prevalence of vitamin D deficiency among the South Asian adults: A systematic review and meta-analysis. BMC Public Health.

[48] Smirnova D.V., Rehm C.D., Fritz R.D., Kutepova I.S., Soshina M.S., Berezhnaya Y.A. (2022). Vitamin D status of the Russian adult population from 2013 to 2018. Sci. Rep..

[49] Wu Z., Wu Y., Rao J., Hu H., Wang C., Wu J., Shi Y., Fu Y., Cheng X., Li P. (2022). Associations among vitamin D, tobacco smoke, and hypertension: A cross-sectional study of the NHANES 2001–2016. Hypertens. Res..

[50] Herrick K.A., Storandt R., Afful J., Pfeiffer C.M., Schleicher R.L., Gahche J.J., Potischman N. (2019). Vitamin D status in the United States, 2011–2014. Am. J. Clin. Nutr..

[51] Rooney M.R., Harnack L., Michos E.D., Ogilvie R.P., Sempos C.T., Lutsey P.L. (2017). Trends in Use of High-Dose Vitamin D Supplements Exceeding 1000 or 4000 International Units Daily, 1999–2014. JAMA J. Am. Med. Assoc..

[52] OECD, European Observatory on Health Systems and Policies (2023). Romania: Country Health Profile 2023.

[53] Ministry of Health Ghid Privind Evaluarea Statusului Vitaminei D la Adulti. https://oldsite.ms.ro/wp-content/uploads/2019/07/Anexa2-Ghid-pentru-evaluarea-statusului-vitaminei-D-la-adulti-1-1.pdf.

[54] Ministry of Health Activity Report of the Ministry of Health for 2022, p. 561. https://ms.ro/ro/informatii-de-interes-public/rapoarte/rapoarte-de-activitate/.

[55] Kuulasmaa K., Tolonen H. (2013). What Is EHES and Why It Is Needed?.

[56] Tolonen H., Koponen P., Al-kerwi A.a., Capkova N., Giampaoli S., Mindell J., Paalanen L., Ruiz-Castell M., Trichopoulou A., Kuulasmaa K. (2018). European health examination surveys—A tool for collecting objective information about the health of the population. Arch. Public Health.

[57] Guga Ș., Mihăilescu A., Spatari M. (2018). Coșul Minim de Consum Lunar Pentru un Trai Decent Pentru Populația României.

[58] Institutul National de Sanatate Publica, Home Page-Proiecte in Derulare The Methodology of Implementation of the European Health Survey (EHES) in Romania. https://proiect-pdp1.insp.gov.ro/ancheta-europeana-de-examinare-a-starii-de-sanatate-ehes/.

[59] Souberbielle J.C., Massart C., Brailly-Tabard S., Cavalier E., Chanson P. (2016). Prevalence and determinants of vitamin D deficiency in healthy French adults: The VARIETE study. Endocrine.

[60] Rabenberg M., Scheidt-Nave C., Busch M.A., Rieckmann N., Hintzpeter B., Mensink G.B. (2015). Vitamin D status among adults in Germany--Results from the German Health Interview and Examination Survey for Adults (DEGS1). BMC Public Health.

[61] Bouloukaki I., Markakis M., Pateli R., Lyronis I., Schiza S., Tsiligianni I. (2022). Vitamin D levels in primary care patients: Correlations with clinical, seasonal, and quality-of-life parameters. Fam. Pract..

[62] României C. (2008). Administrația Natională de Meteorologie.

[63] Engelsen O. (2010). The Relationship between ultraviolet radiation exposure and vitamin D status. Nutrients.

[64] Zugravu C.A., Soptica F., Tarcea M., Cucu A. (2016). Pertinence of vitamin D supplementation in the adult Romanian population. Farmacia.

[65] Haq A., Svobodová J., Imran S., Stanford C., Razzaque M.S. (2016). Vitamin D deficiency: A single centre analysis of patients from 136 countries. J. Steroid Biochem. Mol. Biol..

[66] da Silveira E.A., Moura L., Castro M.C.R., Kac G., Hadler M., Noll P., Noll M., Rezende A.T.O., Delpino F.M., Oliveira C. (2022). Prevalence of Vitamin D and Calcium Deficiency and Insufficiency in Women of Childbearing Age and Associated Risk Factors: A Systematic Review and Meta-Analysis. Nutrients.

[67] Rostami M., Tehrani F.R., Simbar M., Bidhendi Yarandi R., Minooee S., Hollis B.W., Hosseinpanah F. (2018). Effectiveness of Prenatal Vitamin D Deficiency Screening and Treatment Program: A Stratified Randomized Field Trial. J. Clin. Endocrinol. Metab..

[68] Hollis B.W., Johnson D., Hulsey T.C., Ebeling M., Wagner C.L. (2011). Vitamin D supplementation during pregnancy: Double-blind, randomized clinical trial of safety and effectiveness. J. Bone Miner. Res. Off. J. Am. Soc. Bone Miner. Res..

[69] Anagnostis P., Livadas S., Goulis D.G., Bretz S., Ceausu I., Durmusoglu F., Erkkola R., Fistonic I., Gambacciani M., Geukes M. (2023). EMAS position statement: Vitamin D and menopausal health. Maturitas.

[70] Danese V.C., Pepe J., Ferrone F., Colangelo L., De Martino V., Nieddu L., Ferrazza G., Panzini E., Pascone R., Blocki F. (2023). The Mutual Interplay between Bone, Glucose and Lipid Metabolism: The Role of Vitamin D and PTH. Nutrients.

